# Identification of key ferroptosis genes and mechanisms associated with breast cancer using bioinformatics, machine learning, and experimental validation

**DOI:** 10.18632/aging.205459

**Published:** 2024-01-19

**Authors:** Shuang Liang, Yan-Ming Bai, Bo Zhou

**Affiliations:** 1 Department of Yinchuan Traditional Chinese Medicine Hospital, Ningxia Medical University, Yinchuan 750001, China; 2 School of Traditional Chinese Medicine, Ningxia Medical University, Yinchuan 750004, China; 3 Ningxia Regional Key Laboratory of Integrated Traditional Chinese and Western Medicine for Prevention and Treatment of High Incidence, Ningxia Medical University, Yinchuan 750004, China

**Keywords:** breast cancer (BC), ferroptosis, bioinformatics, machine learning, *in vitro* experiments

## Abstract

Objective: The aim of this paper is to mine ferroptosis genes associated with breast cancer based on bioinformatics and machine learning, and to perform *in vitro* functional validation.

Methods: Transcriptional and clinical data of breast cancer patients were downloaded from TCGA database and ferroptosis-related genes were obtained from FerrDB database. Significant differentially expressed ferroptosis-related genes between breast cancer tissues and adjacent normal tissues were selected. Functional enrichment analysis was performed on these differentially expressed genes. Four machine learning algorithms were used to identify key ferroptosis-related genes associated with breast cancer. A multi-factor Cox regression analysis was used to construct a risk score model for the key ferroptosis-related genes. The accuracy of the risk score model was validated using Kaplan-Meier survival curve analysis and receiver operating characteristic (ROC) curve analysis. Finally, cell experiments were conducted to validate the biological functions of the key ferroptosis-related genes in breast cancer cells MCF-7, further confirming the accuracy of the analysis results.

Results: A total of 52 significantly differentially expressed ferroptosis-related genes were identified, which were mainly enriched in cancer pathways, central carbon metabolism in cancer, HIF-1 signaling pathway, and NOD-like receptor signaling pathway. Three key ferroptosis-related genes (TXNIP, SLC2A1, ATF3) closely related to the occurrence, development, and prognosis of breast cancer were identified using machine learning algorithms. The risk model constructed using these three key ferroptosis-related genes showed that the prognosis of the low-risk group was better than that of the high-risk group (*P* < 0.001). The ROC curve analysis showed that the prognosis model had good predictive ability. *In vitro* experiments validated the reliability of the bioinformatics and machine learning screening results. Downregulation of SLC2A1 expression promoted ferroptosis and suppressed tumor cell growth in breast cancer cells (*P* < 0.01), while overexpression of TXNIP or ATF3 had the same effect (*P* < 0.01).

Conclusion: This study identified three key ferroptosis-related genes (TXNIP, SLC2A1, ATF3) associated with breast cancer, which are closely related to the occurrence, development, and prognosis of breast cancer.

## INTRODUCTION

Breast cancer (BC) is a prevalent and predominantly female tumor disease that can be classified into different subtypes based on molecular features, histopathological characteristics, and clinical outcomes. Based on the expression levels of hormone receptors (estrogen receptor, progesterone receptor) and HER2/neu (human epidermal growth factor receptor 2), breast cancer can be classified into three subtypes, including hormone receptor-positive (ER+ or PR+)/HER2-negative (HER2-) subtype, hormone receptor-negative (ER- and PR-)/HER2-positive (HER2+) subtype, and hormone receptor-negative/HER2-negative (triple-negative) subtype. Breast cancer has a high incidence and mortality rate [1] with approximately 1.6 million new cases and a mortality rate of around 6.4% globally each year [2, 3] with approximately 1.6 million new cases and a mortality rate of around 6.4% globally each year [4]. Despite significant advances in breast cancer treatment in recent years, the mortality rate remains high, and many patients are diagnosed and treated late due to the lack of effective methods for early diagnosis and screening [4]. The pathogenesis of breast cancer is complex and multifactorial, involving genetic and environmental factors. Many genes, such as the tumor suppressor genes BRCA1 and BRCA2, have been found to be closely associated with the occurrence and development of breast cancer [5]. Approximately 75% of breast cancer patients have mutations or low expression of BRCA1 or BRCA2 [5]. Therefore, exploring the genes and proteins that are abnormally expressed during the occurrence and development of breast cancer, studying the molecular characteristics involved in breast cancer, and identifying new prognostic biomarkers are of great significance for better understanding of its pathological mechanism, developing new effective diagnostic and therapeutic methods, and improving clinical outcomes.

Ferroptosis is a novel form of programmed cell death characterized by the imbalance of redox status and iron metabolism in cells. Due to the high energy demand of tumor cells, they exhibit high levels of oxidative reactions and obvious iron addiction compared to normal cells, with high levels of iron death markers, such as reactive oxygen species (ROS) and ferrous ions (Fe^2+^) [6]. Studies have shown that inducing ferroptosis of breast cancer cells is an important approach to improve or treat breast cancer [7, 8]. Many clinical drugs such as metformin [7], simvastatin [9], lapatinib [10], etc., can improve the occurrence, development, and prognosis of breast cancer by inducing ferroptosis. Thus, exploring ferroptosis-related genes closely associated with breast cancer is of great significance for studying its pathogenesis, diagnosis, treatment, and prognosis assessment.

In this study, we applied bioinformatics and machine learning methods to screen ferroptosis-related genes closely associated with breast cancer and verified their functions through *in vitro* experiments. This study will further enrich our understanding of the role of ferroptosis in the occurrence, development, and prognosis of breast cancer and provide a basis for the molecular mechanism study, diagnosis, treatment, and prognosis assessment of breast cancer.

## MATERIALS AND METHODS

### Data download and preprocessing

Transcriptome data and clinical information related to breast cancer were obtained from The Cancer Genome Atlas (TCGA) database (https://portal.gdc.cancer.gov). A total of 1204 transcriptome data samples were included, consisting of 1091 breast cancer tumor tissue samples and 113 adjacent normal tissue samples. The clinical information contained 1097 breast cancer patients. After excluding breast cancer patients without transcriptome data and breast cancer patients with overall survival time less than 10 days, clinical data of 1060 breast cancer patients were selected for prognostic analysis. Table 1 shows the statistical description of the clinical data of 1060 breast cancer patients. The transcriptome data were processed as follows: the Ensemble ID in the transcriptome dataset was converted to gene names based on the gene annotation information in the TCGA database, and a new expression matrix was generated by extracting the protein-coding genes in the dataset. The expression matrix was then normalized and standardized. A total of 259 ferroptosis-related genes were obtained from the FerrDB database (http://www.zhounan.org/ferrdb/).

**Table 1:** Clinical characteristics of the 1060 breast cancer patients from TCGA database.

**Characteristics**	**Alive (*N* = 913)**	**Dead (*N* = 147)**	**Total (*N* = 1060)**
**Gender**			
Female	902 (85.09%)	146 (13.77%)	1048 (98.87%)
Male	11 (1.04%)	1 (0.09%)	12 (1.13%)
**Histological type**			
Infiltrating Carcinoma NOS	1 (0.10%)	0 (0%)	1 (0.10%)
Infiltrating Ductal Carcinoma	653 (66.09%)	104 (10.53%)	757 (76.62%)
Infiltrating Lobular Carcinoma	177 (17.91%)	22 (2.23%)	199 (20.14%)
Medullary Carcinoma	3 (0.30%)	2 (0.20%)	5 (0.51%)
Metaplastic Carcinoma	7 (0.71%)	1 (0.10%)	8 (0.81%)
Mucinous Carcinoma	14 (1.42%)	3 (0.30%)	17 (1.72%)
Not Available	1 (0.10%)	0 (0%)	1 (0.10%)
**Pathologic stage**			
Stage I	165 (15.73%)	16 (1.53%)	181 (17.25%)
Stage II	537 (51.19%)	63 (6.01%)	600 (57.20%)
Stage III	195 (18.59%)	43 (4.10%)	238 (22.69%)
Stage IV	4 (0.38%)	15 (1.43%)	19 (1.81%)
Stage X	5 (0.48%)	6 (0.57%)	11 (1.05%)
**Pathologic T**			
T1	245 (23.11%)	33 (3.11%)	278 (26.23%)
T2	538 (50.75%)	75 (7.08%)	613 (57.83%)
T3	106 (10.00%)	24 (2.26%)	130 (12.26%)
T4	22 (2.08%)	14 (1.32%)	36 (3.40%)
TX	2 (0.19%)	1 (0.09%)	3 (0.28%)
**Pathologic N**			
N0	455 (42.92%)	43 (4.06%)	498 (46.98%)
N1	295 (27.83%)	59 (5.57%)	354 (33.40%)
N2	97 (9.15%)	21 (1.98%)	118 (11.13%)
N3	58 (5.47%)	15 (1.42%)	73 (6.89%)
NX	8 (0.75%)	9 (0.85%)	17 (1.60%)
**Pathologic M**			
M0	760 (72.11%)	117 (11.10%)	877 (83.21%)
M1	4 (0.38%)	17 (1.61%)	21 (1.99%)
MX	143 (13.57%)	13 (1.23%)	156 (14.80%)
**Age**			
Mean ± SD	57.76 ± 12.56	60.47 ± 14.43	58.13 ± 12.86
Median (min-max)	58 (26.00, 89.00)	62.00 (26.00, 89.00)	58.00 (26.00, 89.00)
**Overall survival time**			
Mean ± SD	1210.05 ± 1158.61	1617.74 ± 1309.93	1266.59 ± 1188.48
Median (min-max)	791.00 (010.00, 8605.00)	1174.00 (30.00, 7455.00)	876.50 (10.00, 8605.00)

### Materials and reagents

The human breast cancer cell line MCF-7 was purchased from the Cell Bank of the Chinese Academy of Sciences. DMEM medium and fetal bovine serum were purchased from Gibco (Grand Island, NY, USA); Op-timen medium was purchased from Procell Life Science and Technology (Wuhan, China); Lipofectamine 2000 transfection reagent was purchased from Invitrogen (Thermo Fisher Scientific, Carlsbad, CA, USA). Small interfering RNA targeting SLC2A1 (siRNA-SLC2A1), pcDNA-TXNIP overexpression plasmid (TXNIP-OE), and pcDNA-ATF3 overexpression plasmid (ATF3-OE) were designed and synthesized by Sangon Biotech (Shanghai, China); SLC2A1, TXNIP, and ATF3 primers were synthesized by Sangon Biotech (Shanghai, China). Primary antibodies against SLC2A1 (#4393), TXNIP (#13113), and ATF3 (#12691) were purchased from Abcam (Cambridge, UK), and anti-actin was purchased from Proteintech Group (Wuhan, China). HRP-conjugated secondary antibody was purchased from Elabscience Biotechnology (Wuhan, China). BCA protein assay kit was purchased from Elabscience Biotechnology (Wuhan, China). TRIzol reagent, PrimeScript RT reagent Kit, and SYBR Green Master Mix were purchased from Thermo Fisher Scientific (Wilmington, NC, USA). Reactive oxygen species (ROS) and Fe^2+^ detection assay kits were purchased from Yeasen Biotechnology (Shanghai, China), glutathione (GSH) detection assay kits were purchased from Beijing Solarbio Science and Technology (Beijing, China), malondialdehyde (MDA) assay kits were purchased from Nanjing Jiancheng Biological Engineering Research Institute (Nanjing, China). CCK-8 assay kit was purchased from New Cell and Molecular Biotech (Suzhou, China). GIEMS staining solution was purchased from Gibco (Grand Island, NY, USA).

### Identification of differentially expressed genes associated with ferroptosis

Differential expression analysis of transcriptome data was performed using the R package “limma”. A fold change threshold of 2.5 and a *P* < 0.05 were set to identify differentially expressed genes (DEGs) between tumor (BC group, *n* = 1091) and adjacent tissues (NC group, *n* = 113). To further investigate the relationship between DEGs and ferroptosis, the identified DEGs were compared with ferroptosis-related genes downloaded from the FerrDB database (http://www.zhounan.org/ferrdb). By overlapping the two gene sets, a list of ferroptosis-related DEGs was identified, which may play critical roles in breast cancer.

### Functional enrichment analysis

To further investigate the biological functions and pathways associated with ferroptosis, the differentially expressed genes (DEGs) related to ferroptosis were input into the DAVID database (https://david.ncifcrf.gov/) for functional enrichment analysis. Gene Ontology (GO) annotation and Kyoto Encyclopedia of Genes and Genomes (KEGG) pathway analysis were performed to identify GO terms and KEGG pathways that were significantly enriched (*P* < 0.05). The results of the functional enrichment analysis were visualized using R software. The identified enriched GO terms and KEGG pathways could provide insights into the molecular mechanism of ferroptosis in breast cancer.

### Identification of key ferroptosis-related genes associated with breast cancer by machine learning

Four machine learning algorithms, namely Maximal Clique Centrality (MCC), Random Forest, Support Vector Machine Recursive Feature Elimination (SVM-RFE), and LASSO Regression, were employed to screen ferroptosis-related genes associated with the development, progression, and prognosis of breast cancer. The MCC algorithm was used to explore genes that potentially play a central regulatory role among these ferroptosis-related DEGs by calculating the topological parameters of the nodes in the PPI network. First, the STRING database (http://string-db.org) was utilized to perform Protein-Protein Interaction (PPI) analysis on the ferroptosis-related DEGs. After PPI network construction, the MCC module in the CytoHubba plugin in Cytoscape 3.7.1 software was used to calculate MCC value for each node. The nodes were ranked by MCC value and the top 20 were selected. Random Forest and SVM-RFE algorithms are commonly used effective algorithms for identifying potential disease biomarkers. In this study, R packages “randomForest” was utilized to construct the random forest model with the following key parameters: the number of trees (ntree) was set to 500, the number of variables randomly sampled as candidates at each split (mtry) was set to the default value of square root of the number of features. Based on the feature importance scores assigned by the model, all the ferroptosis-related genes were ranked in descending order of importance. The SVM-RFE approach was implemented using the “e1071” package in R. A linear kernel function was specified for the SVM classifier. Recursive feature elimination was carried out by iteratively removing the features with the smallest weights based on the fitted SVM model. After recursive elimination, the remaining ferroptosis-related genes were ranked in ascending order according to their weights assigned by the final SVM model. Moreover, by integrating clinical information of breast cancer patients and expression matrix of ferroptosis-related genes in tumor samples, LASSO regression was performed using the R package “rms” to identify ferroptosis-related genes closely associated with the prognosis of breast cancer patients. LASSO regression performs feature selection and model building by penalizing the model with L1 regularization to force irrelevant coefficients to zero. This process yields a feature subset with non-zero coefficients. Finally, the common ferroptosis-related genes identified under the four machine learning algorithms were considered as key ferroptosis-related genes associated with the development, progression, and prognosis of breast cancer.

### Construction of prognostic risk model based on the key ferroptosis-related genes

To investigate the association between key ferroptosis-related genes and breast cancer prognosis, multivariate Cox regression analysis was employed to construct prognostic risk model. The risk score for each patient was calculated according to the following formula:


$$\text{Risk Score}=\sum_{i=0}^{n}Coef_{i}\times exp_{i}$$

(n represents the number of genes composing the prognostic model, Coef is the risk coefficient of each prognostic gene, and exp represents the standardized expression level of each prognostic gene). Based on the risk scores of each patient, all tumor samples were classified into high-risk (*n* = 530) and low-risk (*n* = 530) groups. The R package “survival” was used to plot Kaplan-Meier survival curves, comparing the overall survival rates of high-risk and low-risk groups. Subsequently, Receiver Operating Characteristic (ROC) analysis was performed and ROC curves were plotted to evaluate the accuracy of this prognostic model for predicting the survival time of breast cancer patients.

### Cell culture and transfection

The breast cancer cell line MCF-7 was cultured in high-glucose DMEM supplemented with 10% FBS and 1% penicillin-streptomycin at 37°C and 5% CO_2_ in a cell culture incubator. Cells in logarithmic growth phase were digested with trypsin and seeded into 6-well plates at an appropriate density. After the cells grew to approximately 60% confluency, Lipofectamine 2000 transfection reagent was used to transfect siRNA-SLC2A1, TXNIP overexpression plasmid, and ATF3 overexpression plasmid mixed with Opti-MEM medium into MCF-7 cells. Empty plasmids (siRNA-NC and NC-OE) were also transfected into MCF-7 cells as negative controls. After incubating for 6 h in the cell culture incubator, the medium was replaced, and the transfection efficiency was detected by q-PCR and Western blotting 24 h later. The cells were divided into four groups according to the treatment: normal control group (Control), SLC2A1 knockdown group (siRNA-SLC2A1), TXNIP overexpression group (TXNIP-OE), and ATF3 overexpression group (ATF3-OE). The cells in each group were used for subsequent experiments 24 h after transfection.

### Quantitative reverse transcription-PCR (q-PCR)

First, total RNA was extracted from the transfected cells and normal cells using the TRIzol reagent according to the manufacturer’s instructions. The RNA concentration and purity were determined using a NanoDrop spectrophotometer (Applied Thermo Fisher Scientific, Wilmington, NC, USA). Then, cDNA was synthesized from the extracted RNA using the PrimeScript RT reagent Kit according to the manufacturer’s instructions. Finally, q-PCR was performed using the SYBR Green PCR Master Mix on the ABI 7500 Real-Time PCR System (Applied Biosystems, Wilmington, USA). The primers utilized in q-PCR are detailed in Table 2.

**Table 2:** Primer sequences for q-PCR assay.

**Gene**	**Forward primer (5′–3′)**	**Reverse primer (5′–3′)**	**Product size (bp)**
SLC2A1	5′-GGCTTCTCCAACTGGACCTC-3′	5′-CCGGAAGCGATCTCATCGAA-3′	176
TXNIP	5′-ATGCCACCCAAGCATTCCTTA-3′	5′-AGGAAGCTCAAAGCCGAACT-3′	153
ATF3	5′-GGAGTGCCTGCAGAAAGAGT-3′	5′-CCATTCTGAGCCCGGACAAT-3′	147
β-actin (ACTB)	5′-ACACAGTGCTGTCTGGTGG-3′	5′-CAGAGTACTTGCGCTCAGGA-3′	129

### Western blot

To validate the expression of ferroptosis-related genes in breast cancer cell line MCF-7, Western blot analysis was performed. Briefly, after transfection with siRNA-SLC2A1, TXNIP overexpression plasmid, and ATF3 overexpression plasmid, respectively, the cells were harvested and lysed using RIPA buffer containing protease inhibitor cocktail. The protein concentration was determined by the BCA protein assay kit. Equal amounts of protein were separated by SDS-PAGE and transferred onto PVDF membranes. After blocking with 5% non-fat milk, the membranes were incubated with primary antibodies against the selected ferroptosis-related genes and β-actin (loading control) at 4°C overnight. After washing with TBST, the membranes were incubated with HRP-conjugated secondary antibodies at room temperature for 1 h. The protein bands were visualized using ECL reagents and analyzed using ImageJ software. The relative expression levels of the ferroptosis-related genes were normalized to β-actin.

### Cell proliferation assay

To assess cell proliferation, single-cell suspensions of the Control group and experimental groups (siRNA-SLC2A1, TXNIP-OE, ATF3-OE) were seeded into 96-well plates at a density of approximately 2000 cells per well and incubated at 37°C and 5% CO_2_ in a cell culture incubator for 24, 48, and 72 h. At each time point, 10 μL of CCK8 working solution was added to each well, and the cells were incubated for 30 min at 37°C. The absorbance (A) was measured at 450 nm wavelength according to the instructions of the CCK8 kit.

### Colony formation assay

To evaluate colony formation ability, cells from each group were digested with trypsin and made into single-cell suspensions. Approximately 1000 cells were seeded into each well of a 6-well plate and cultured at 37°C and 5% CO_2_ in a cell culture incubator for 21 days, with the medium changed every 3 days. After incubation, the cells were stained with GIEMS solution for 15 min and photographed under a microscope.

### Detection of cell ferroptosis

The levels of GSH, MDA, and ferrous iron ions (Fe^2+^) in cells were measured according to the instructions of the detection kit. First, the transfected cells were cultured for 24 h, and then collected and lysed using RIPA cell lysis buffer containing 1% protease and phosphatase inhibitor. The lysates were centrifuged at 13000 × g for 5 min, and the supernatant was collected. The cell lysates were mixed with the assay reagents, and the absorbance was measured using a microplate reader. The detection wavelengths for GSH, MDA, and Fe^2+^ were 412 nm, 532 nm, and 593 nm, respectively. Furthermore, the reactive oxygen species (ROS) levels in cells were measured according to the instructions of a ROS detection kit. Transfected cells were cultured for 24 h and collected to prepare a single-cell suspension. A suitable amount of 10 μmol/L DCFH-DA probe solution was added to the cells, and they were incubated at 37°C for 30 min. After washing the cells twice with serum-free cell culture medium, the fluorescence intensity was measured using a flow cytometer with an excitation wavelength of 488 nm and an emission wavelength of 525 nm.

### Statistical analysis

Statistical analysis was performed using GraphPad Prism 8.3.0 software. All experimental data were repeated at least three times and presented as mean ± standard deviation (SD). Differences between groups were compared using the independent sample *t*-test and one-way analysis of variance (ANOVA). A *p*-value less than 0.05 was considered statistically significant.

### Availability of data and materials

The data sets supporting the results of this article are included within the article. All data, models, and code generated or used during the study appear in the submitted article.

## RESULTS

### Screening of differentially expressed genes associated with ferroptosis

**Figure 1:**
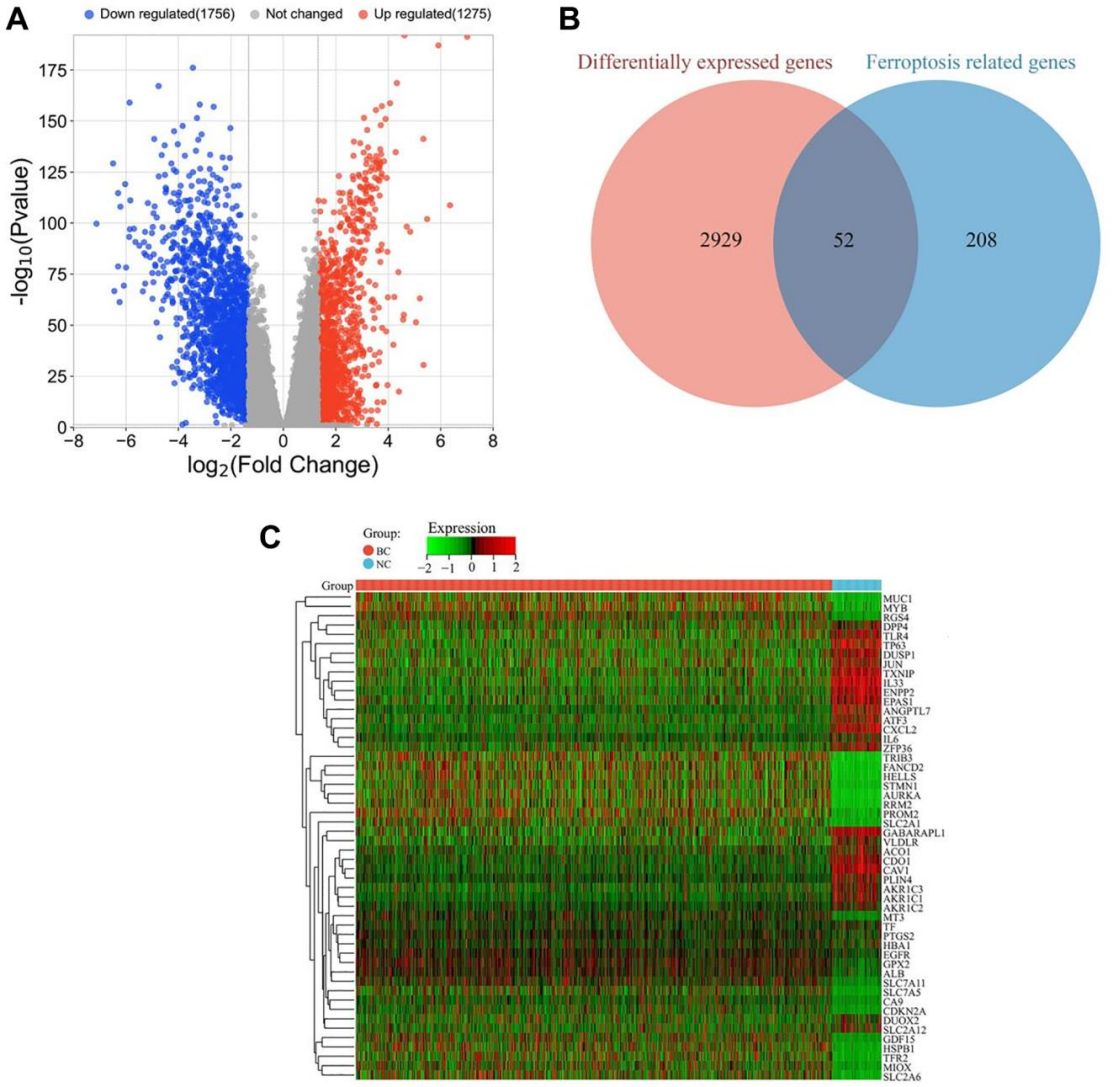
**Identification of ferroptosis-related DEGs in breast cancer.** (**A**) Volcano plot of all DEGs between tumor (BC group, *n* = 1091) and adjacent tissues (NC group, *n* = 113). Red and blue dots represent significantly upregulated and downregulated genes, respectively. (**B**) Venn diagram showing the overlap between ferroptosis-related genes and DEGs. (**C**) Heatmap of differentially expressed ferroptosis-related genes in tumor (BC group, *n* = 1091) and adjacent tissues (NC group, *n* = 113). The color scale represents the relative expression level of each gene, with red indicating high expression and green indicating low expression.

A total of 3031 differentially expressed genes (DEGs) were identified between tumor (BC group) and adjacent tissues (NC group), including 1275 significantly upregulated genes and 1756 significantly downregulated genes (Figure 1A). To further investigate the relationship between DEGs and ferroptosis, the identified DEGs were compared with ferroptosis-related genes downloaded from the Ferroptosis Database (FerrDB). By overlapping the two gene sets, a list of 52 ferroptosis-related DEGs was identified, which may play critical roles in breast cancer (Figure 1B). The expression levels of these ferroptosis-related DEGs are presented in a heatmap (Figure 1C).

### Functional enrichment analysis

**Figure 2:**
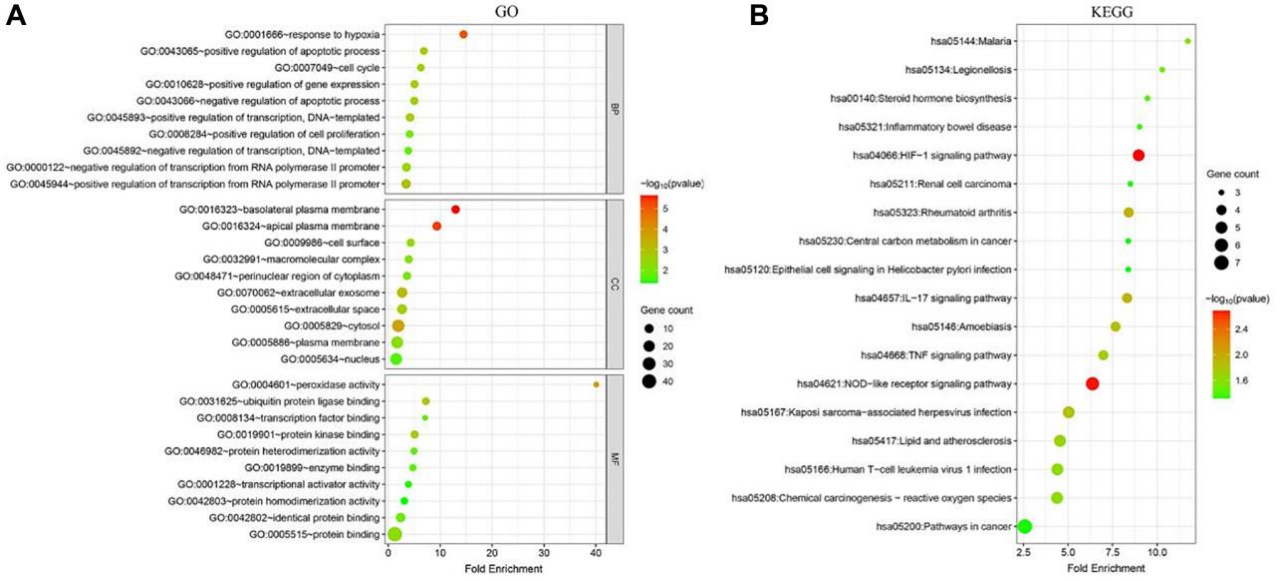
**Functional enrichment analysis of ferroptosis-related DEGs in breast cancer.** (**A**) Gene Ontology (GO) analysis of ferroptosis-related DEGs. The top 10 enriched terms in each category (biological process, cellular component, and molecular function) are shown. (**B**) Kyoto Encyclopedia of Genes and Genomes (KEGG) pathway analysis of ferroptosis-related DEGs. The top 10 enriched pathways are shown.

The GO analysis showed that the ferroptosis-related DEGs were mainly involved in biological process (BPs) such as positive regulation of transcription from RNA polymerase II promoter, negative regulation of transcription from RNA polymerase II promoter, and response to hypoxia, as well as cellular components (CCs) such as cytosol, plasma membrane, nucleus, and extracellular exosome. In addition, they were enriched in molecular functions (MFs) such as identical protein binding, protein kinase binding, ubiquitin protein ligase binding, and protein homodimerization activity. These results suggest that the ferroptosis-related DEGs may play important roles in the regulation of transcription, hypoxia response, and protein-protein interactions (Figure 2A).

The KEGG pathway analysis revealed that the ferroptosis-related DEGs were significantly enriched in several cancer-related pathways, including Pathways in cancer, Central carbon metabolism in cancer, HIF-1 signaling pathway, and NOD-like receptor signaling pathway. These pathways are known to be associated with cancer development and progression, suggesting that the ferroptosis-related DEGs may play important roles in breast cancer (Figure 2B).

### Screening of key ferroptosis-related genes

**Figure 3:**
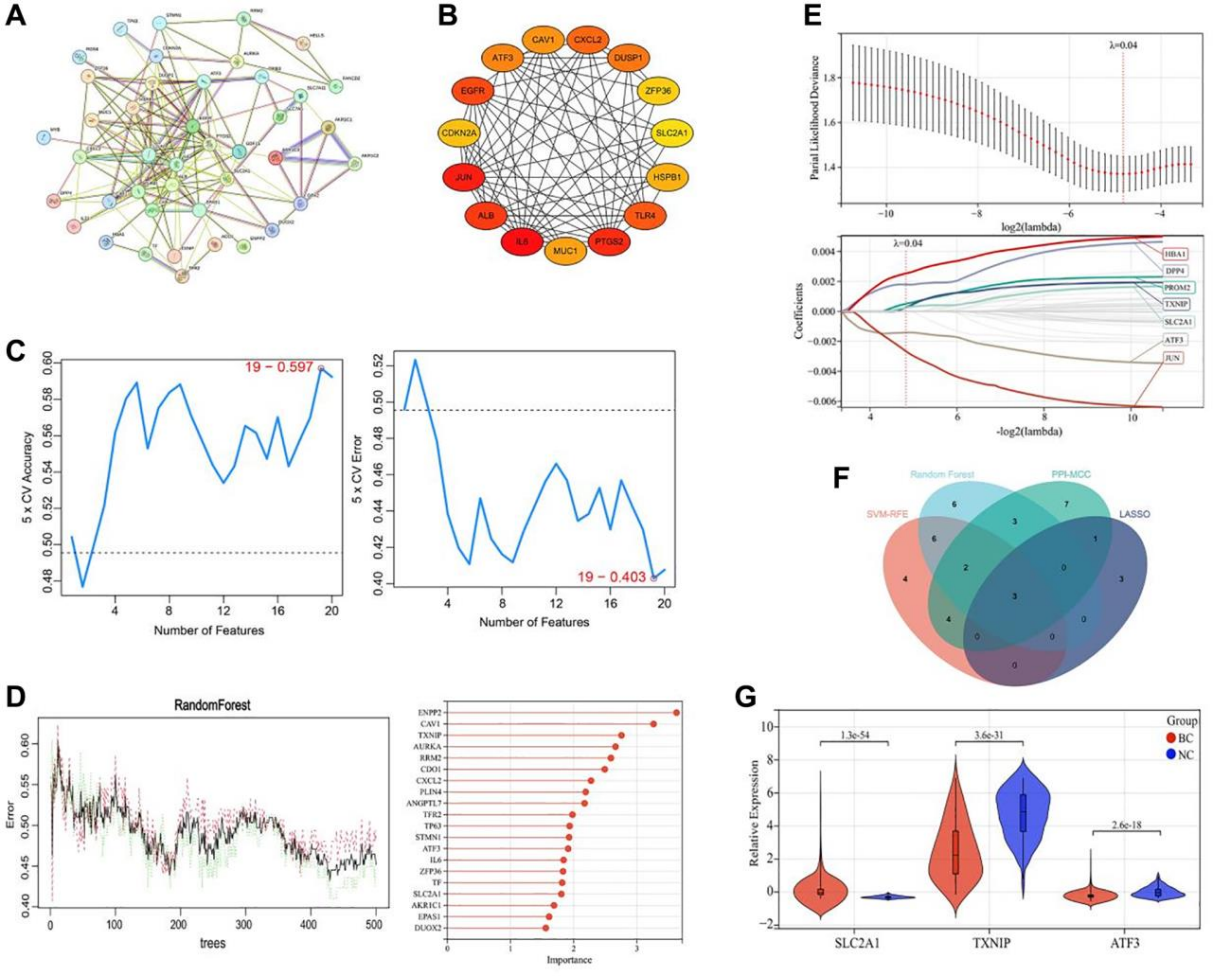
**Screening of key ferroptosis-related genes in breast cancer.** (**A**) Protein-protein interaction network of ferroptosis-related DEGs. (**B**) Top 20 genes with the best connectivity identified by the MCC algorithm. (**C**) SVM-REF analysis of the feature ferroptosis-related genes for differentiating breast cancer tissues from adjacent tissues. (**D**) Random forest analysis of the feature ferroptosis-related genes for differentiating breast cancer tissues from adjacent tissues. (**E**) LASSO regression analysis of the ferroptosis-related genes associated with the prognosis of breast cancer patients. (**F**) Venn diagram showing the common ferroptosis-related genes identified by the 4 machine learning algorithms. (**G**) Expression levels of TXNIP, SLC2A1, and ATF3 in breast cancer tissues (BC group, *n* = 1091) and adjacent tissues (NC group, *n* = 113).

The 52 ferroptosis-related DEGs were analyzed by constructing a PPI network, which revealed that they interacted with each other (Figure 3A). Based on the maximal clique centrality (MCC) algorithm, we identified the top 20 genes with the best connectivity from the PPI network by calculating the topological parameters of the nodes (Figure 3B). SVM-REF analysis showed that a model containing 19 ferroptosis-related genes had the highest accuracy in differentiating breast cancer tissues from adjacent tissues (Figure 3C). The top 20 genes identified by random forest analysis were selected as the feature ferroptosis-related genes for differentiating breast cancer tissues from adjacent tissues (Figure 3D). In addition, LASSO regression analysis identified 7 ferroptosis-related genes that were closely associated with the prognosis of breast cancer patients (Figure 3E). By integrating the results of the 4 machine learning algorithms, we identified 3 common ferroptosis-related genes, namely TXNIP, SLC2A1, and ATF3 (Figure 3F). Among them, TXNIP and ATF3 were significantly downregulated in breast cancer tissues compared to adjacent tissues (*P* < 0.001), while SLC2A1 was significantly upregulated in breast cancer tissues compared to adjacent tissues (*P* < 0.001) (Figure 3G).

### Construction of the prognostic risk model

**Figure 4:**
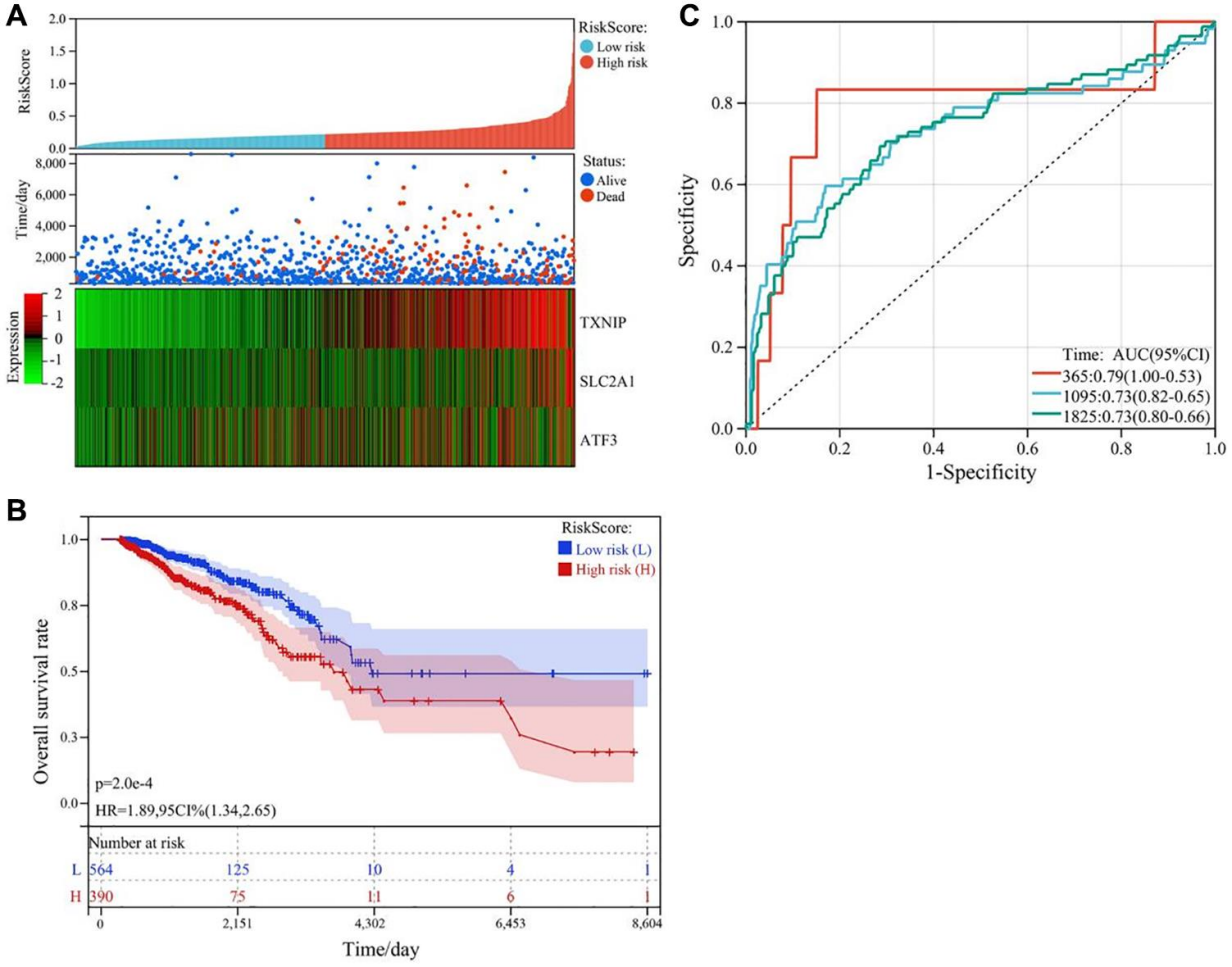
**Construction and validation of the ferroptosis-related gene prognostic risk model in breast cancer.** (**A**) Survival status plot of breast cancer patients based on the prognostic model. (**B**) Kaplan-Meier curve analysis of the overall survival rate of high-risk (*n* = 530) and low-risk (*n* = 530) groups. (**C**) ROC curve analysis of the prognostic model for predicting the survival of breast cancer patients at 365 days, 1095 days, and 1825 days. The AUC values were 0.79, 0.73, and 0.73, respectively.

We used multivariate Cox regression analysis to construct a risk score model, which was calculated as follows: RiskScore = SLC2A1 × 0.813354160440223 − TXNIP × 0.473133422604816 − ATF3 × 0.21764547803389237. The survival status plot of breast cancer patients based on the prognostic model is shown in Figure 4A. Based on the risk score of each patient, all tumor samples were divided into high-risk and low-risk groups. The results showed that the overall survival rate of the high-risk group was significantly lower than that of the low-risk group (*P* < 0.001), as shown in Figure 4B. ROC curve analysis validated the good predictive ability of the prognostic model for survival. The AUC values of the prognostic model for predicting the survival of breast cancer patients at 365 days, 1095 days, and 1825 days were 0.79, 0.73, and 0.73, respectively, as shown in Figure 4C.

### Functional validation of key ferroptosis-related genes in breast cancer cells

**Figure 5:**
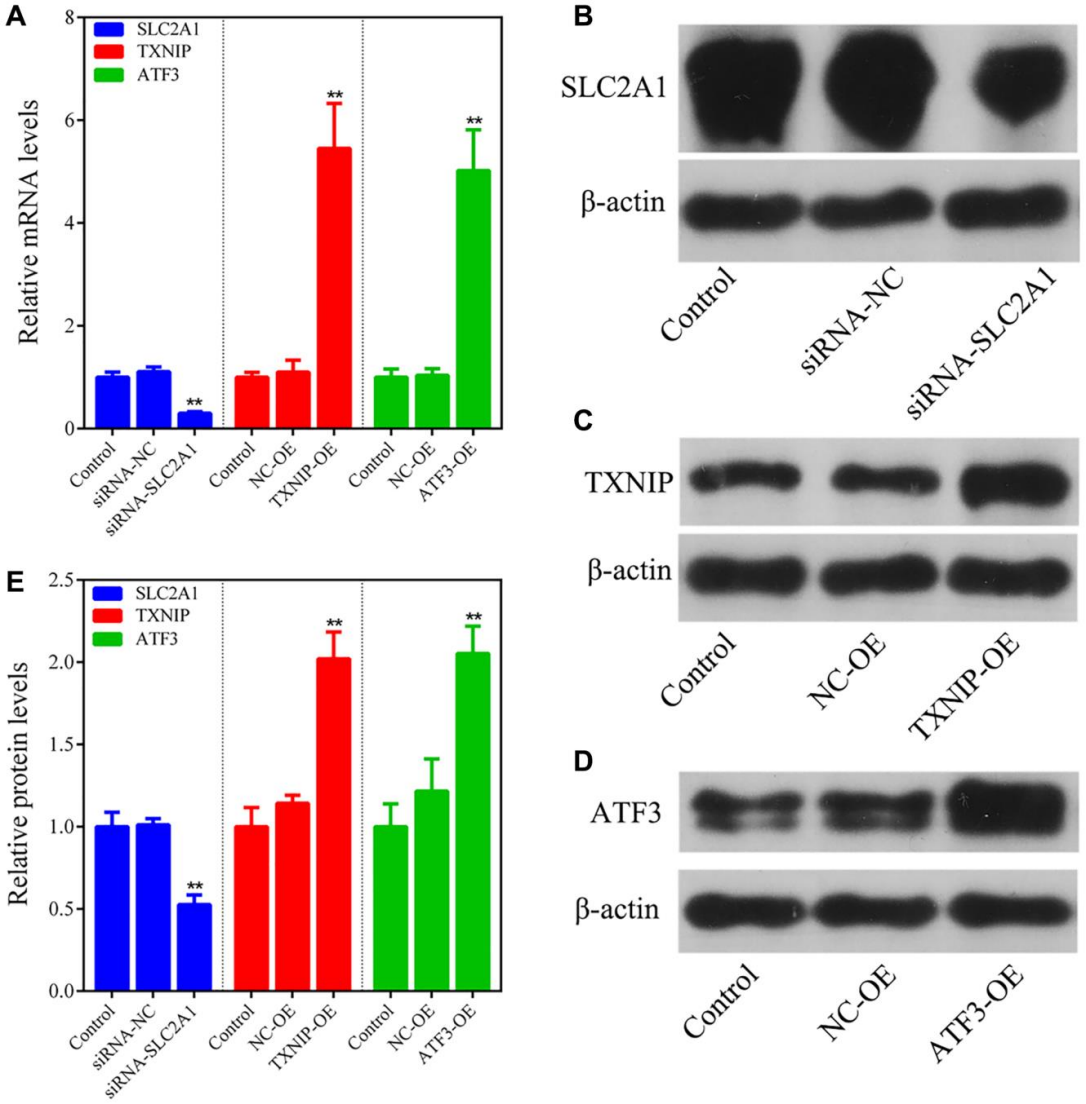
**Transfection efficiency detection.** (**A**) q-PCR analysis of the transfection efficiency of siRNA-SLC2A1, TXNIP overexpression plasmids, and ATF3 overexpression plasmids. (**B**–**E**) Western blot analysis of the transfection efficiency of siRNA-SLC2A1, TXNIP overexpression plasmids, and ATF3 overexpression plasmids. The bar graphs show the mean ± SD of at least three independent experiments (*n* = 3). ^**^P < 0.01, compared to the Control group, the difference is extremely significant.

We conducted *in vitro* transfections with siRNA-SLC2A1, TXNIP overexpression plasmids, and ATF3 overexpression plasmids to further investigate the effects of key ferroptosis-related genes on ferroptosis in breast cancer cells. Transfection results showed that compared to the normal control group (Control), SLC2A1 mRNA and protein levels were significantly downregulated in the SLC2A1 knockdown group (siRNA-SLC2A1) (*P* < 0.05), while TXNIP mRNA and protein levels were significantly upregulated in the TXNIP overexpression group (TXNIP-OE) (*P* < 0.05), and ATF3 mRNA and protein levels were significantly upregulated in the ATF3 overexpression group (ATF3-OE) (*P* < 0.05) (Figure 5A–5E). These results indicate that the transfection efficiency was good in all experimental groups.

**Figure 6:**
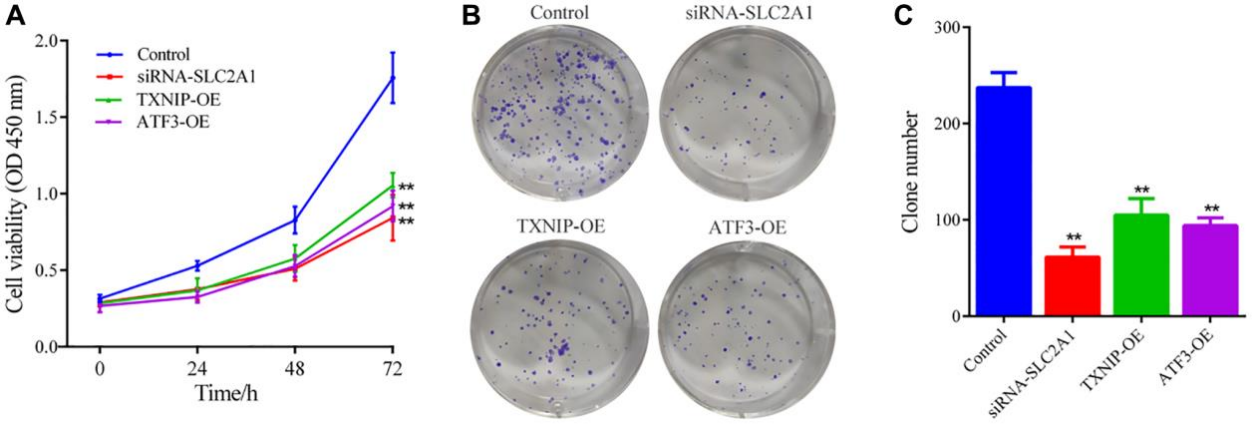
**Cell growth ability detection.** (**A**) CCK8 assay was used to detect the effects of SLC2A1, TXNIP, and ATF3 on the cell viability of MCF-7 cells. (**B**, **C**) Colony formation assay was used to detect the effects of SLC2A1, TXNIP, and ATF3 on the colony formation ability of MCF-7 cells. The data presented are the mean ± SD of at least three independent experiments (*n* = 3). ^**^P < 0.01, compared to the Control group, the difference is extremely significant.

Cell growth was assessed using CCK8 and colony formation assays. CCK8 results showed that at 24, 48, and 72 hours, cell viability in all experimental groups (siRNA-SLC2A1, TXNIP-OE, and ATF3-OE) was significantly decreased compared to the normal control group (Control) (*P* < 0.05) (Figure 6A). The results of the colony formation assay showed that the number of cell colonies in all experimental groups was significantly reduced compared to the normal control group (Control) (*P* < 0.05) (Figure 6B, 6C). These results indicate that downregulation of SLC2A1 or upregulation of TXNIP or ATF3 expression can significantly inhibit the growth of breast cancer cells.

**Figure 7:**
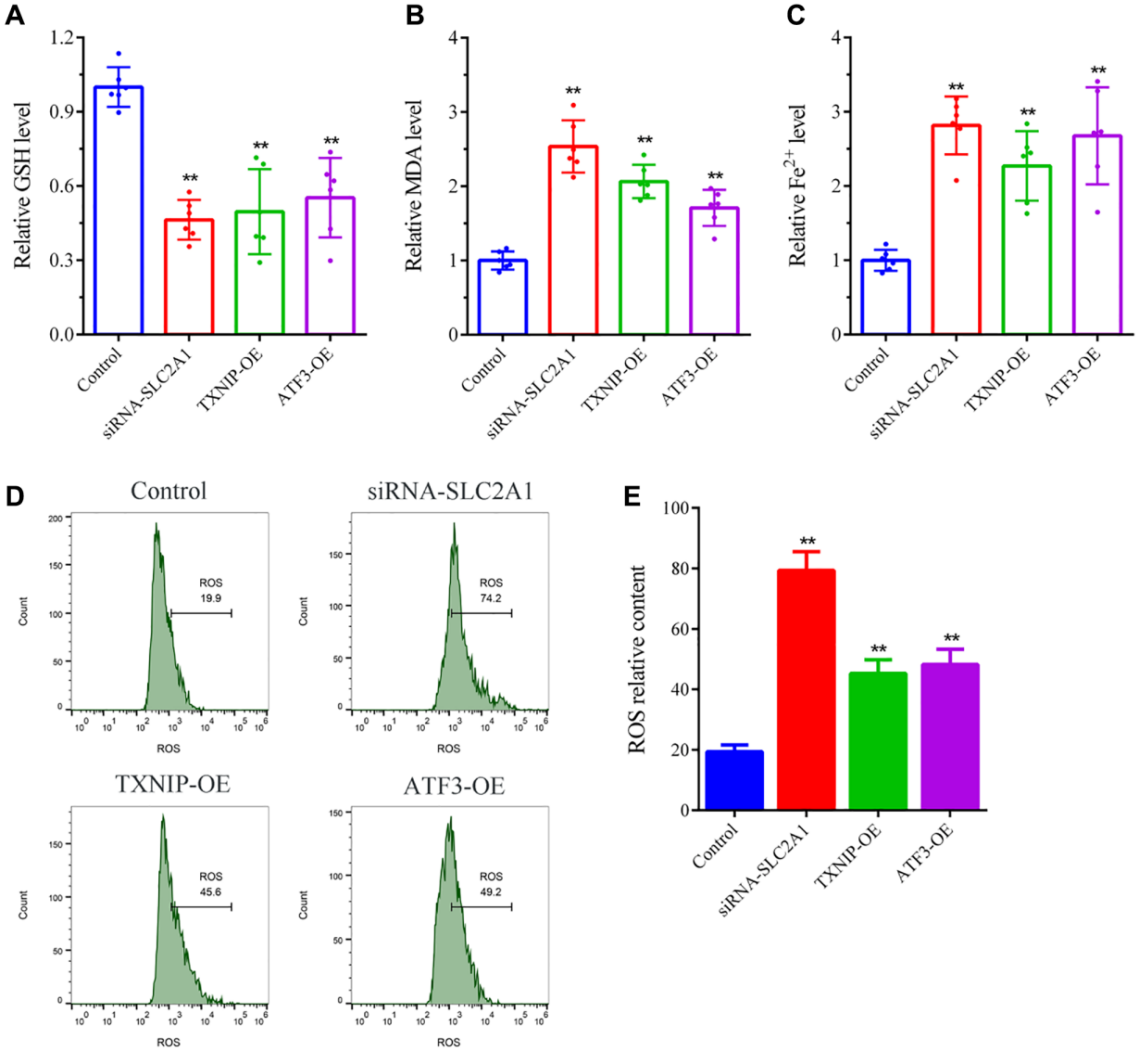
**Ferroptosis detection in breast cancer cells.** (**A**) GSH levels detection. (**B**) MDA levels detection. (**C**) Fe^2+^ levels detection. (**D**, **E**) ROS levels detection. The bar graphs show the mean ± SD of at least three independent experiments (*n* = 3). ^**^P < 0.01, compared to the Control group, the difference is extremely significant.

To investigate the effects of key ferroptosis-related genes (TXNIP, SLC2A1, and ATF3) on ferroptosis in breast cancer cells, we measured the levels of GSH, MDA, and Fe^2+^ in the cells. The results showed that compared to the normal control group (Control), GSH, Fe^2+^, and ROS levels were significantly reduced (*P* < 0.05), and MDA levels were significantly increased (*P* < 0.05) in the experimental groups (siRNA-SLC2A1, TXNIP-OE, and ATF3-OE) (Figure 7A–7E). These results indicate that downregulation of SLC2A1 or upregulation of TXNIP or ATF3 expression can induce ferroptosis in breast cancer cells.

## DISCUSSION

Breast cancer is the most common cancer among women, with a complex and multifactorial pathogenesis that remains unclear. Despite advances in diagnosis and treatment, breast cancer still accounts for a significant proportion of cancer-related deaths. Therefore, there is an urgent need to identify novel approaches for early diagnosis, effective treatment, and accurate prognosis evaluation. Recent studies have highlighted the critical role of ferroptosis in breast cancer development and progression. Many ferroptosis-related genes have been found to be closely related to the growth of breast cells. Cystine/glutamate transporter (SLC7A11), encoded by the SLC7A11 gene, is a multi-channel transmembrane protein that promotes GSH synthesis and reduces ROS to inhibit cell ferroptosis by transporting cystine into cells and exchanging intracellular glutamate [11, 12]. Inhibition of SLC7A11 expression and methylation levels can increase lipid ROS levels and induce ferroptosis in breast cancer cells, thus inhibiting tumor growth [7]. Phospholipid hydroperoxide glutathione peroxidase (GPX4) is a ferroptosis inhibitory protein that converts lipid hydroperoxides into alcohols using reduced GSH to alleviate cell oxidative damage [13]. It was found that GPX4 is highly expressed in breast cancer tissues and its high expression is closely associated with poor prognosis of breast cancer patients [14]. Inhibition of GPX4 expression in breast cells can trigger ferroptosis, a form of iron-dependent cell death, which can suppress tumor cell proliferation and induce cell apoptosis [15].

Integrative analysis of expression profiling data using bioinformatics methods is one of the most effective approaches for identifying disease pathogenesis, biomarkers, and prognostic features, which has the advantages of low cost and high efficiency. Machine learning is an artificial intelligence method that uses statistical algorithms applied to datasets by computers and is widely used for feature information screening in high-throughput data. The combination of bioinformatics methods and machine learning provides a more reliable and effective technique for screening disease-associated genes, which has gradually become a technical hotspot in omics research. In this study, bioinformatics and machine learning were combined for research on the ferroptosis mechanism in breast cancer. We performed differential expression analysis on breast cancer-related datasets from the TCGA database, and identified 52 DEGs associated with ferroptosis in cancer and adjacent tissues. Functional analysis revealed that these genes are involved in multiple signaling pathways closely related to breast cancer progression, such as the HIF-1 and NOD-like receptor signaling pathways. Previous studies have shown that the HIF-1 pathway can influence breast cancer growth by regulating tumor cell metabolism, the Wnt/β-catenin signaling pathway, angiogenesis, and other pathways [16–19]. Activation of the NOD-like receptor pathway can promote estrogen receptor signaling and ROS production, which further inhibits breast cancer cell growth [20]. The NOD-like receptor pathway is also an important pathway for activating the body’s immune signal, promoting the secretion of inflammatory factors such as TNF-α, and further killing breast cancer cells [21]. Additionally, the HIF-1 and NOD-like receptor pathways can regulate cell ferroptosis [22, 23], although their association with ferroptosis in breast cancer cells has not been reported.

We utilized four machine learning algorithms to identify three key ferroptosis-related genes (TXNIP, SLC2A1, ATF3) that are closely associated with the development and prognosis of colon cancer from 52 differentially expressed genes. A prognostic model was constructed using these three genes, and the results showed that high expression of SLC2A1 is associated with poor prognosis in breast cancer patients, while low expression of TXNIP and ATF3 is associated with poor prognosis. The accuracy of this prognostic model was further validated through Kaplan-Meier survival curve analysis and ROC curve analysis. Thioredoxin-interacting protein (TXNIP), also known as thioredoxin-binding protein-2, is a widely expressed protein in various tissues and organs [24]. TXNIP negatively regulates the activity of thioredoxin (TXN) by binding to it, leading to oxidative stress, mitochondrial dysfunction and cell death [25]. As an inducer of ferroptosis, TXNIP causes iron accumulation and accumulation of lipid ROS by inhibiting the activity of GSH, GPX4, and other redox enzymes, ultimately inducing cell death [26]. TXNIP has great potential as a therapeutic target for breast cancer, as studies have shown that it is a good biomarker and prognostic evaluation gene for breast cancer. Park et al. showed that TXNIP expression was reduced in breast cancer patients and that TXNIP downregulation activated estrogen receptor signaling, which in turn enhanced the proliferative activity of breast cancer cell [27]. Downregulation of TXNIP can promote glucose uptake and Warburg effect, while forced overexpression of TXNIP can inhibit these processes [28]. Chen et al. showed that TXNIP can inhibit the proliferation of TNBC resistant cells and promote apoptosis by increasing ROS synthesis and DNA damage caused by doxorubicin, thus reducing TNBC resistance to chemotherapy [29]. In addition, studies have shown that TXNIP expression is downregulated in other tumors such as hepatocellular carcinoma, lung cancer and bladder cancer. TXNIP can inhibit tumor cell growth and metastasis by suppressing metabolic reprogramming and promoting oxidative stress [30]. Activating transcription factor 3 (ATF3) is a stress-induced transcription factor (e.g., by DNA damage, oxidative stress, and cellular injury) that plays an important role in the progression of breast cancer and other tumor diseases. Low expression of ATF3 is closely related to poor prognosis in breast cancer patients [31]. Upregulating ATF3 expression in breast cancer cells can inhibit their growth, migration, and invasion [31]. Research has found that ATF3 regulates transcription by binding to the promoter sequences of multiple oncogenes and tumor suppressor genes, exerting anti-tumor effects [32]. Furthermore, ATF3 can induce ferroptosis by inhibiting the Xc^−^ amino acid antiporter to deplete intracellular GSH and promote the production of lipid peroxides, leading to cell death [33]. ATF3 can also bind to the promoter of ferroptosis-related gene SLC7A11 and induce cell death by suppressing its expression [33]. Solute carrier family 2 member 1 (SLC2A1), also known as glucose transporter 1 (GLUT1), is the earliest discovered glucose transporter. SLC2A1 is highly expressed in breast cancer and other tumor diseases and is closely associated with the progression and metastasis of various cancers [34, 35]. Studies have found that SLC2A1 can resist ferroptosis by promoting glycolysis [36]. Inhibition of SLC2A1 can suppress the glycolysis process, cell proliferation, migration, and metastasis of breast cancer cells [1]. Additionally, inhibition of SLC2A1 can increase autophagic flux to suppress tamoxifen resistance in breast cancer cells [37]. In summary, the three key ferroptosis-related genes (TXNIP, SLC2A1, ATF3) are closely associated with the development and prognosis of breast cancer. However, it is still unclear whether TXNIP, SLC2A1, and ATF3 affect the growth of breast cancer cells by regulating the ferroptosis mechanism.

To validate the reliability of combining bioinformatics and machine learning analysis, we employed *in vitro* gene silencing and overexpression techniques to observe the effects of SLC2A1 knockdown, TXNIP upregulation, and ATF3 upregulation on the growth of breast cancer cells and ferroptosis-related indicators. The results showed that consistent with the results of bioinformatics and machine learning analysis, SLC2A1 is a breast cancer risk gene, and its knockdown can induce iron death of breast cancer cells and inhibit cancer cell growth. TXNIP and ATF3 are tumor suppressor genes in breast cancer, and their upregulation can trigger the ferroptosis mechanism and suppress the growth of breast cancer cells.

## CONCLUSION

This study identified three key ferroptosis-related genes (TXNIP, SLC2A1, ATF3) associated with breast cancer by integrating bioinformatics analysis and multiple machine learning algorithms, and preliminarily validated their roles through cell experiments. The innovation lies in the effective strategy of combining bioinformatics and machine learning to screen key genes. However, this study has some limitations, including lack of *in vivo* validation. Future directions include validating the results in animal models and elucidating the molecular mechanisms. In summary, the study further confirms the important role of the ferroptosis mechanism in the occurrence, development, and treatment of breast cancer, providing new insights for the study of the pathogenesis, diagnosis, and prognosis evaluation of breast cancer.
